# ESR Essentials: bone marrow MRI in oncology—practice recommendations by the European Society of Musculoskeletal Radiology

**DOI:** 10.1007/s00330-025-12307-4

**Published:** 2026-02-09

**Authors:** Frédéric E. Lecouvet, Lokmane Taihi, Thomas Kirchgesner, Vassiliki Pasoglou, Marin Halut, Hatice Tuba Sanal, Salvatore Gitto, Teodoro Martín-Noguerol, Violeta Vasilevska-Nikodinovska, Filip Vanhoenacker, Joan C. Vilanova

**Affiliations:** 1 Department of Medical Imaging, Institut de Recherche Expérimentale et Clinique (IREC), Institut du Cancer Roi Albert II, Cliniques Universitaires Saint Luc, Université Catholique de Louvain (UCL), Brussels, Belgium; 2 Department of Radiology, Centre Hospitalier Universitaire Sainte-Justine, Montreal, QC Canada; 3 Department of Radiology, Centre Hospitalier de l’Université de Montréal (CHUM), Montréal, QC Canada; 4 Department of Radiology, Radiation Oncology and Nuclear Medicine, Université de Montréal, Montréal, QC Canada; 5 University of Health Sciences, Gülhane Training and Research Hospital, Ankara, Türkiye; 6 Dipartimento di Scienze Biomediche per la Salute, Università degli Studi di Milano, Milan, Italy; 7 IRCCS Istituto Ortopedico Galeazzi, Milan, Italy; 8 MRI Section, Radiology Department, HT medica, Jaén, Spain; 9 University “Ss.CYril and Methodius”, Skopje, Macedonia; 10 University Surgical Clinic “St.Naum Ohridski”, Skopje, Macedonia; 11 General Hospital Sint-Maarten, Mechelen, Belgium; 12 Department of Radiology, University Hospital Antwerp, Edegem, Belgium; 13 Faculty of Medicine and Health Sciences, University of Antwerp, Ghent and Leuven, Antwerpen, Belgium; 14 Department of Radiology, Clinica Girona, Institute of Diagnostic Imaging (IDI) Girona, University of Girona, Girona, Spain

**Keywords:** Bone, Bone marrow, Neoplasms, Therapeutics, Treatment outcome

## Abstract

**Abstract:**

Involvement of the bone marrow by metastases from solid tumors or multiple myeloma (MM) is a critical challenge in oncologic imaging. Lesion detection and staging, as well as accurate assessment of treatment response, disease recurrence, and complications, are key to optimal patient management. This article provides recommendations for performing and interpreting bone marrow MRI in cancer patients. MRI should be the primary imaging modality for patients suspected of having skeletal bone metastases or MM, and should replace radiography, bone scintigraphy, and CT for these indications. Protocols must be tailored to the clinical context and to each specific cancer. Whole-body MRI (WB-MRI) is preferred for a comprehensive assessment, while axial skeleton MRI (AS-MRI) is a fast and reliable alternative for targeted or follow-up evaluations. We recommend standardized protocols that incorporate anatomical sequences (preferably fast spin echo T2 Dixon) and diffusion-weighted imaging (DWI). Quantitative biomarkers, e.g., apparent diffusion coefficient (ADC) and fat fraction (FF), should be implemented to improve diagnostic accuracy and evaluate treatment response. Radiologists must be familiar with the typical patterns of bone marrow replacement by cancer cells, response assessment principles, and common imaging pitfalls. Every medical imaging facility should offer optimal bone marrow MRI and implement these recommendations using available MRI systems and existing disease-oriented guidelines. This ESR Essentials illustrates when, how, and why to perform bone marrow MRI to improve diagnostic precision and oncologic care across a broad range of indications.

**Key Points:**

- *Prefer MRI of the bone marrow over radiographs, bone scintigraphy, or CT for suspected bone metastases of solid cancers and for myeloma staging*.
- *Use MRI for diagnosis of bone involvement, disease staging, assessment of lesion response to treatment, detection of recurrence, and assessment of osseous complications*.
- *Tailor MRI protocol to cancer type following existing guidelines, targeting either the axial skeleton or the “whole body,” and using a panel of sequences with fat-sensitive, fast spin echo T2 Dixon and diffusion-weighted sequences as the fundamental components*.

## Key Recommendations

- Use bone marrow MRI as the first-line imaging modality in patients with suspected bone metastasis of solid tumors or multiple myeloma. Prefer MRI over radiographs, bone scintigraphy, and computed tomography (CT), based on superior diagnostic performance (level of evidence: high; prospective cohort studies, systematic reviews, international guidelines).
- Apply MRI for comprehensive assessment of bone marrow involvement for disease staging, assessment of treatment response, evaluation of residual disease, detection of recurrence, and work-up of complications (level of evidence: high; prospective cohort studies, systematic reviews, international guidelines).
- Tailor MRI protocols to cancer type and clinical scenario, using standardized sequences including fast spin echo T2 Dixon and diffusion-weighted imaging (DWI), and quantitative information such as the apparent diffusion coefficient (ADC) and fat fraction (FF). Protocols should cover either the axial skeleton or the whole body. Follow disease-specific guidelines for appropriate acquisition, interpretation, and reporting (level of evidence: high; validated international guidelines).

## Introduction

Bone metastases from solid tumors and multiple myeloma (MM) are the most common secondary and primary bone malignancies in adults, respectively. Accurate assessment of skeletal involvement in these conditions is essential for proper staging, evaluation of treatment response, detection of residual disease, and identification of recurrence.

MRI, whether focused on the axial skeleton (AS-MRI) or performed as whole-body MRI (WB-MRI), is established as a reliable alternative to traditional imaging techniques such as radiographs, bone scintigraphy, and computed tomography (CT) [1]. WB-MRI is recommended alongside positron-emission tomography combined with computed tomography (PET/CT) in many cancers for staging and assessment of treatment response.

While PET/CT is largely performed and allows for high-performance disease work-up when adapted radiopharmaceuticals exist, WB-MRI has become the first-line imaging modality in various malignancies, having the advantage of tracer independence and absence of radiation [2, 3].

Although WB-MRI can assess both skeletal and extra-skeletal disease, this paper focuses specifically on bone marrow involvement.

This ESR Essentials provides practical recommendations for performing and interpreting bone marrow MRI in patients with metastatic solid cancer and MM. The recommendations are endorsed by the European Society of musculoSkeletal Radiology (ESSR) and are designed to assist general radiologists in delivering optimized oncologic care.

An example of a report template is proposed to help radiologists in their report on the technique, findings of the bone marrow study and assessment of lesion response (Supplementary Material).

## History and bone marrow MRI and its role among oncologic imaging modalities

The introduction of MRI in the 1980s revolutionized oncologic imaging by enabling direct, radiation-free assessment of the bone marrow, transforming the diagnostic management of many cancers by offering direct insight into a previously inaccessible body compartment.

Unlike radiographs, bone scintigraphy, and CT, which only detect indirect or delayed signs of skeletal involvement, MRI offers early and sensitive detection of marrow replacement. MRI protocols have evolved to assess the axial skeleton MRI (AS-MRI) and, later, to whole-body MRI (WB-MRI) [1]. The integration of diffusion-weighted imaging (DWI) has added functional information, enhancing detection and treatment monitoring capabilities. Multiple trials and meta-analyses have demonstrated the superiority of MRI over conventional imaging for detecting bone metastases and multiple myeloma (MM) [4]. Importantly, it allows for the quantitative evaluation of treatment response or progression in lesions previously considered non-measurable [5]. MRI is now recommended alongside PET/CT for the staging and assessment of treatment response, with the advantage of tracer independence and absence of radiation [6].

**Table 1:** Flowchart summarizes the oncological indications of bone marrow MRI along the care pathway

Clinical stage	Indications and principles of imaging
Newly diagnosed cancer and multiple myeloma	Follow the clinico-biological evaluation of the risk of bone involvement Use MRI for early detection and staging of skeletal disease Choose whole-body MRI (WB-MRI) (exhaustive approach) or axial skeleton MRI (AS-MRI) (fast alternative)
↓	
Treatment response monitoring	Compare with the same examination obtained at baseline Combine the morphological and functional assessment of the response Use lesion size and number, fat fraction (FF) and apparent diffusion coefficient (ADC) values
↓	
End of treatment	Assess possible residual disease using MRI, preferably WB-MRI Establish post-treatment baseline Identify treatment-related changes
↓	
Disease recurrence	Use MRI facing clinical or biochemical suspicion of recurrence Perform MRI for systematic surveillance in high-risk patients Compare to the end of treatment study
+	
Assessment of skeletal complications	Evaluate suspected pathologic fractures, spinal cord compression Use targeted MRI sequences Complement with CT if necessary for assessing bone structure/stability

*WB-MRI* whole-body MRI, *AS-MRI* axial skeleton MRI, *ADC* apparent diffusion coefficient

## Indications of bone marrow MRI in oncology (Table 1)

Bone marrow MRI plays a central role across the disease continuum in cancer patients. At diagnosis, clinicians identify cancer patients who should benefit from systematic bone screening, especially those with cancers known to frequently metastasize to bone or who present with specific biological, histological or cytogenetic risk factors. In MM, MRI is required to support accurate initial staging. During therapy, MRI allows monitoring of treatment response. After treatment, MRI is performed to identify residual disease and establish a baseline for long-term surveillance. MRI allows the detection of recurrence, the work-up of complications, and the identification of treatment-related effects.

## Target cancers and recommended imaging approaches

The paper focuses on bone metastases and MM, the two most studied and guideline-recommended indications of bone marrow MRI. Other indications, like lymphoma or leukemia, are not covered as imaging can be challenging or because they still require large-scale validation [7].

### Metastatic cancers

**Fig. 1:**
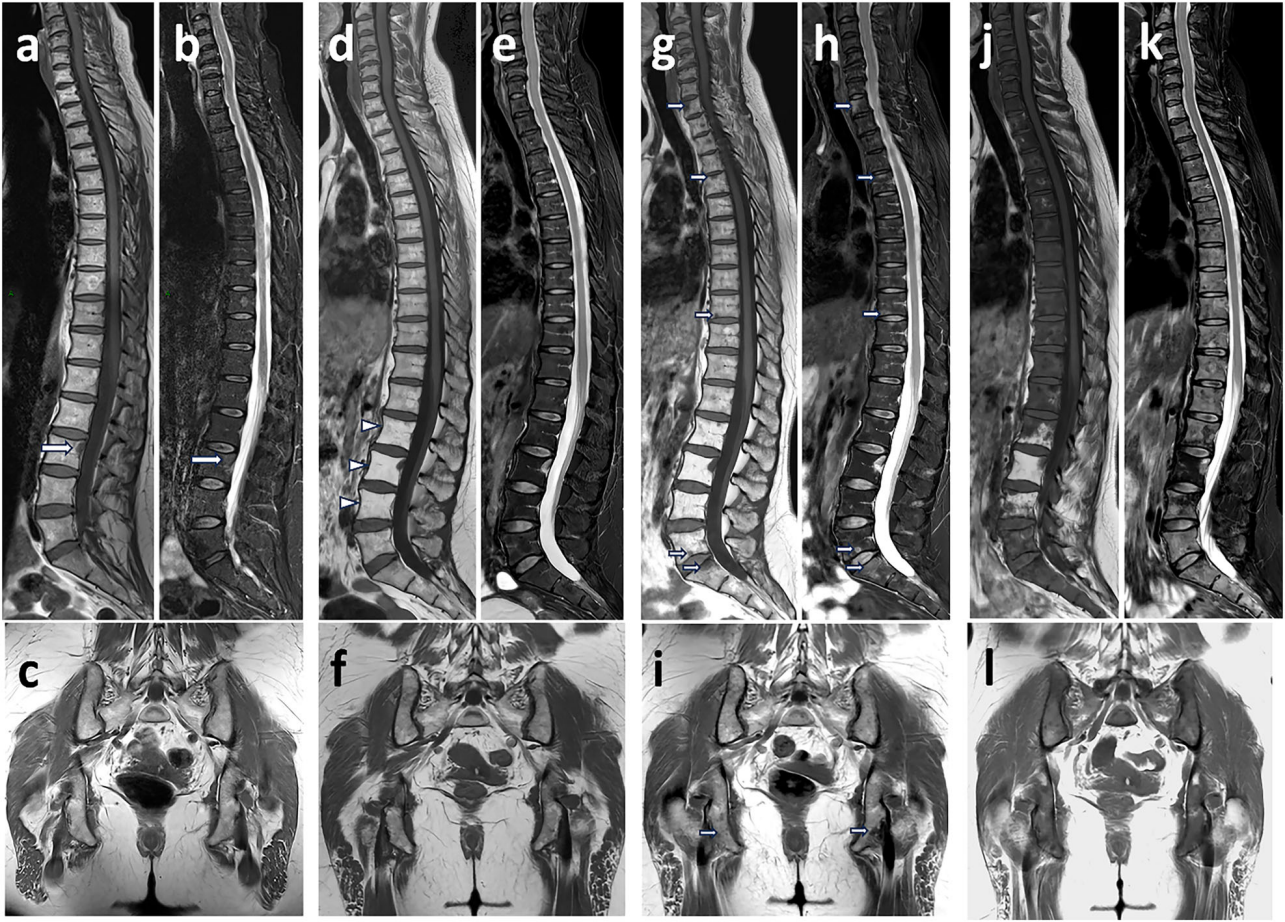
MRI of the axial skeleton (AS-MRI) in a 67-year-old woman with advanced breast cancer; four consecutive AS-MRI studies illustrate initial cancer staging, treatment and follow-up. **a**–**c** AS-MRI study performed at diagnosis, including sagittal T1 (**a**) and T2 Dixon water-only (**b**) images of the spine, and coronal T1-weighted images (**c**) of the pelvis. A small vertebral metastasis is seen within the L3 vertebral body (arrows in **a**, **b**). No pelvic metastasis is present at this time. **d**–**f** Three-month follow-up AS-MRI shows a subtle decrease in the size of the L3 lesion (arrow in **d**). Increase in the signal of the adjacent marrow of L1-L3 on the T1 image (arrowheads in **d**) is related to initiation of targeted radiation therapy to the L3 “oligometastasis,” in complement to systemic treatment. **g**–**i** Twelve-month follow-up AS-MRI shows stability of the L3 lesion and high signal intensity of the adjacent irradiated marrow. Multiple new vertebral and pelvic metastases are observed, representing multifocal metastatic dissemination (small arrows). **j**–**l** Twenty-month follow-up AS-MRI shows stability of the L3 lesion and high signal intensity of the adjacent irradiated marrow. Diffuse low signal on T1 (lower than disks and muscles) and high signal on T2 of the spinal and pelvic bone marrow indicate diffuse metastatic dissemination

Use bone marrow MRI for managing cancers with bone-predominant or bone-exclusive metastatic spread, such as prostate and certain breast cancers [8, 9]. AS-MRI may suffice in these indications to confirm marrow involvement and ensure follow-up during treatment (Fig. 1). It can be combined with contrast-enhanced thoraco-abdomino-pelvic CT to detect node, lung and visceral metastases. WB-MRI is a reliable alternative in this indication.

WB-MRI has been validated in other primary cancers, including lung and colorectal cancers. It surpasses bone scintigraphy and thoraco-abdomino-pelvic CT in accuracy, while reducing examination burden and patient exposure [10, 11].

When choosing between WB-MRI and PET/CT, the availability of a reliable PET tracer is essential. In cancers lacking specific PET/CT tracers or with low [¹⁸F]-FDG avidity, such as lobular breast carcinoma or myxoid liposarcoma, prioritize WB-MRI [12]. In melanoma and neuroendocrine tumors, WB-MRI is a robust alternative to [¹⁸F]-FDG and [⁶⁸Ga]-DOTATOC PET/CT, respectively [13]. In osteosarcoma and Ewing’s sarcoma, WB-MRI plays a key role in evaluating both bone and soft tissue, detecting distant and skip metastases early [14].

Table 2 illustrates the respective strengths and limits of WB-MRI and PET/CT in oncological imaging.

**Table 2:** Strengths and limits of WB-MRI and PET/CT in oncological imaging

Feature	WB-MRI	PET/CT
Tracer dependency	Independent of tracer; provides consistent results for all tissue types (“One size fits all”)	Requires disease-specific tracers. Can miss non-avid lesions (examples: lobular breast cancer metastases and up to 20% of multiple myeloma have no FDG uptake)
Diagnostic value	Higher sensitivity and specificity Works for focal and diffuse marrow lesions	High specificity when cancer-specific tracer exists Less sensitive in diffuse marrow involvement
Response assessment	Morphological and quantitative (number, size, apparent diffusion coefficient (ADC), fat fraction (FF))	Morphological and quantitative (via standardized uptake values (SUVs))
Radiation exposure	No ionizing radiation; preferred for repeated use, young or pregnant patients	Involves ionizing radiation from CT and radionuclide; cumulative exposure is a concern
Anatomical coverage	Whole skeleton and soft tissues, with multi-organ capability	Whole skeleton and soft tissues, with multi-organ capability
Lung assessment	Lower sensitivity for small pulmonary lesions	Higher sensitivity for detecting lung nodules (CT)
Complications/extraosseous findings	Excellent soft tissue characterization Excellent for complications: bone, spinal cord, nerve roots study	Limited anatomical resolution Limited for the detection and pre-therapeutic assessment of complications
Theranostics perspective	Not applicable; cannot guide theranostics	Enables theranostics (e.g., same tracer for imaging and targeted radio-ligand treatment)
Cost	Usually less expensive than PET/CT; reimbursed variably	More expensive; cost varies by tracer and local policies
Availability	Increasing with shortened examination duration	Established in most cancer centers

### Multiple myeloma (MM)

For patients with suspected or confirmed MM, use WB-MRI with DWI as the first-line modality, as endorsed by international guidelines and largely adopted [6]. WB-MRI outperforms radiographs, CT, and even [¹⁸F]-FDG PET/CT in sensitivity [15].

Employ WB-MRI in solitary plasmacytoma, high-risk monoclonal gammopathy of undetermined significance (MGUS) and smoldering myeloma, where positive MRI findings indicate widespread disease, risk for early progression, and need for systemic treatment [16].

**Fig. 2:**
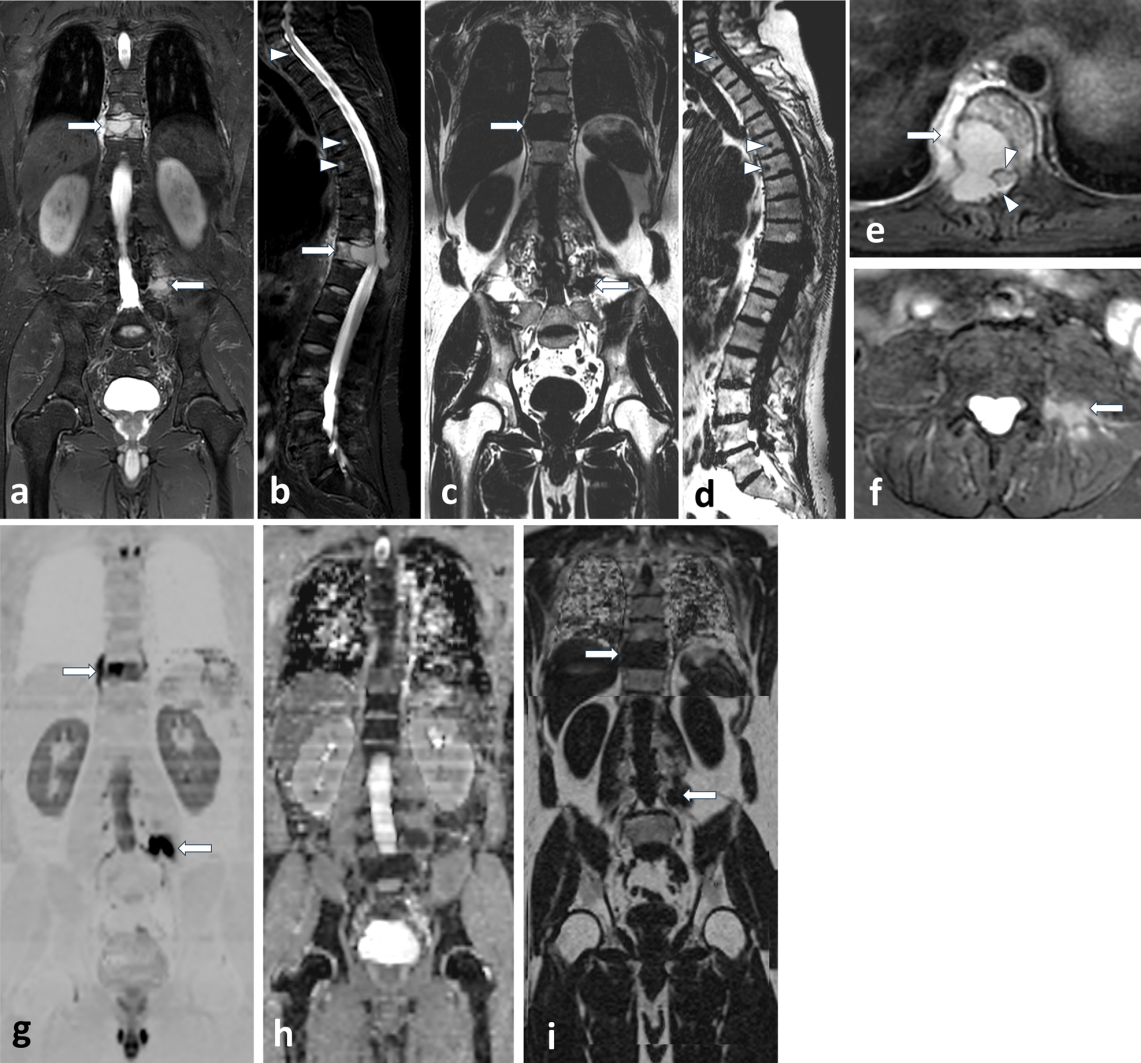
Whole body MRI (WB-MRI) protocol for bone marrow imaging in a 67-year-old patient with newly diagnosed multiple myeloma. **a**–**d** 3D Fast spin echo (FSE) T2 Dixon sequence: water-only (**a**, **b**) and fat-only (**c**, **d**) reformatted coronal and sagittal images obtained from the same isotropic acquisition show two tumoral foci within the T10 vertebral body and left transverse process of L4 (arrows). Smaller foci of low signal intensity on T1 and high signal intensity on T2 images are seen on the sagittal images (arrowheads in **b** and **d**). This sequence also provides in-phase images for visceral study. **e**, **f** Transverse reformatted water-only images show the T10 and L4 lesions (arrows), and most importantly, the canal extension of the T10 focus (arrowheads in **e**). **g**, **h** DWI sequence: reformatted coronal high b-value image (inverted grayscale window, b = 1000 s/mm^2^) shows the T10 and L4 lesions (arrows in **e**). Apparent diffusion coefficient (ADC) map (**f**) is calculated for lesion ADC follow-up after treatment. **i** Fat fraction map (coronal reformat) is also acquired for treatment response assessment; note the visibility of the tumoral foci (arrows)

**Fig. 3:**
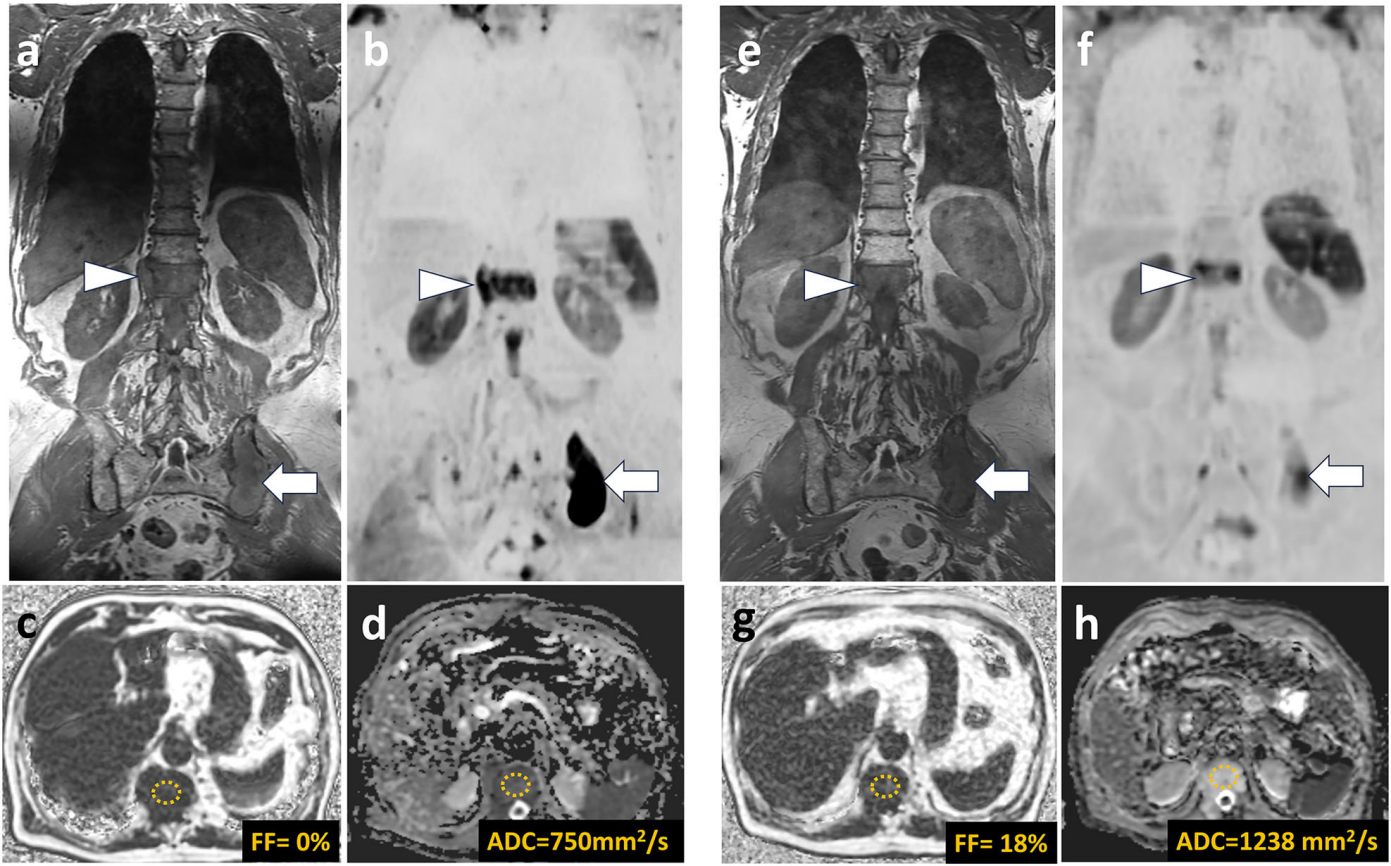
Whole body MRI (WB-MRI) performed at diagnosis and after treatment in a 77-year-old man with multiple myeloma. **a**, **b** Initial coronal T1 (**a**) and high b-value (inverted grayscale window, b = 1000 s/mm^2^) DWI (**b**) sequences show a large lesion within the T12 vertebral body extending into the right paraspinal soft tissues (arrowhead). Another lesion is seen within the left posterior iliac crest (arrow). In addition to the T12 and left iliac lesions, DWI image (**b**) shows a lesion in the right humeral head, which is not described in detail here. The image also shows spots in the pelvis, corresponding to sections of the small bowel, normal-sized lymph nodes, and nerve roots. These are normal findings frequently seen on high b-value DWI images. **c** Transverse fat fraction (FF) map within the T12 body (ROI) shows absence of residual marrow fat (FF = 0%). The iliac lesion FF was 2% (not shown). **d** Transverse Apparent diffusion coefficient (ADC) map within the T12 body (ROI) shows increased ADC compared to normal marrow (750 mm^2^/s), indicating tumoral replacement. The iliac lesion ADC was 765 mm^2^/s (not shown). **e**–**h** Corresponding images and quantitative maps from the WB-MRI examination performed at 2 months follow-up, after systemic treatment and irradiation of the lesions: decrease in soft tissue extension and discrete fading of the lesions is seen on morphologic images (arrowheads and arrows in **e**, **f**). Measurements within the body of T12 show increased FF (18%) (**g**) and ADC (1238 mm^2^/s) (**h**) (ROIs), indicating lesion response to treatment. Measurements within the iliac lesion show increased FF (10%) and ADC (1115 mm^2^/s) (not illustrated)

For response assessment, WB-MRI with DWI is preferable to AS-MRI (Figs. 2, 3). DWI offers higher sensitivity, enables global tumor burden quantification, and distinguishes active disease from inactive post-treatment changes, making it an optimal tool for detecting minimal residual disease (MRD) and recurrence [17].

Although low-dose CT has a high sensitivity to detect osteolytic foci by the time of initial work-up and is recommended in several guidelines, it does not allow for later evaluation of response, which is a major weakness.

## Choice of MRI sequences

Fat-sensitive, fluid-sensitive, and DWI sequences are three pillars offering complementary information for detecting, characterizing, and monitoring disease.

Understanding the normal marrow composition and its evolution with tumoral involvement and during treatment is essential. The adult skeleton houses both red (hematopoietic) and yellow (fatty) marrow. Red marrow, confined to the axial skeleton, contains a 50–60% hematopoietic/hydrated tissue to 40–50% fat ratio, while yellow marrow located in the limbs is > 80% fat. The fat content of the red marrow is critical: tumors replace normal marrow and its fat content, altering signal characteristics [18].

### Recommendation # 1: Use fat-sensitive sequences as the cornerstone of bone marrow MRI

Use T1-weighted and/or Dixon Fat-Only (FO) images to assess marrow integrity [19, 20]. Yellow marrow has a high signal intensity. Red marrow has an intermediate signal intensity because it contains a mixture of fat and hematopoietic cells. Focal or diffuse tumoral replacement of normal marrow results in fat loss and a corresponding decrease in signal intensity, which is lower than that of disks or muscles on T1-weighted images and void of signal on FO Dixon images (Figs. 1–3). In this context, FO Dixon images have shown diagnostic performance comparable to that of T1-weighted images, while offering an increased contrast-to-noise ratio [21]. The reappearance of fat in lesions after treatment indicates a positive response (Fig. 3).

### Recommendation # 2: Use fat-suppressed fluid-sensitive sequences to improve performance and characterization

**Fig. 4:**
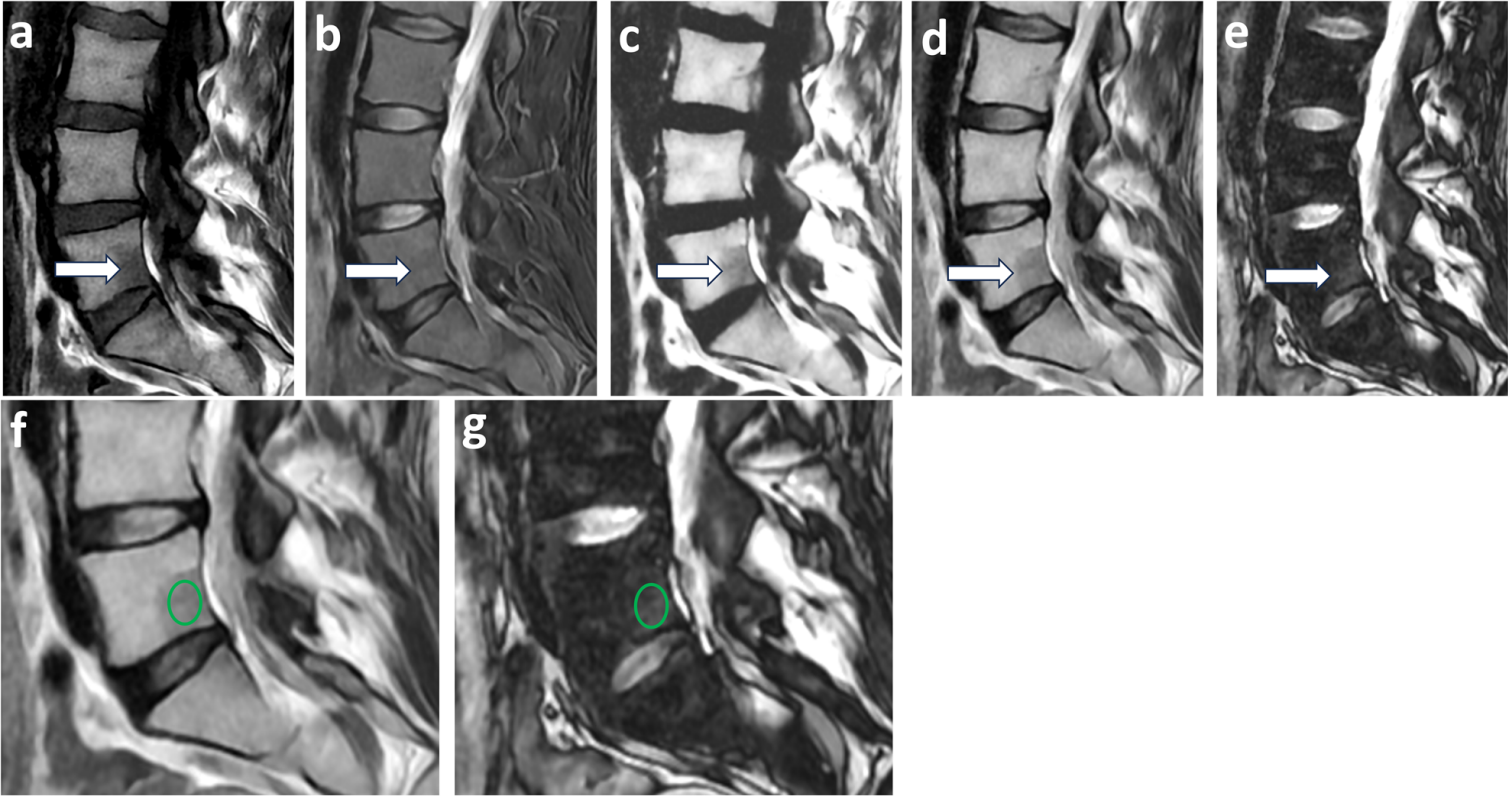
Differential diagnosis between metastasis and benign nodular marrow hyperplasia. Focal marrow hyperplasia in a 42-year-old female with newly diagnosed breast cancer. Sagittal spin echo T1-weighted (**a**), and fast spin echo T2 Dixon water-only (**b**), fat-only (**c**), in-phase (**d**, **f**) and out-phase (**e**, **g**) images. A focal lesion (arrows) shows low signal on T1 (**a**) and relatively low signal on fat Dixon (**c**) images, and intermediate signal on water-only Dixon (**b**). The lesion shows frank signal drop (73%) on the out-phase image (arrow in **e**; ROI with measured signal intensity in **g** = 142) compared to the in-phase image (arrow in **d**; ROI with measured signal intensity in **g** = 540), indicating the presence of fat. These characteristics allow the confident diagnosis of a focus of red bone marrow hyperplasia. The lesion showed no evolution at imaging follow-up

**Fig. 5:**
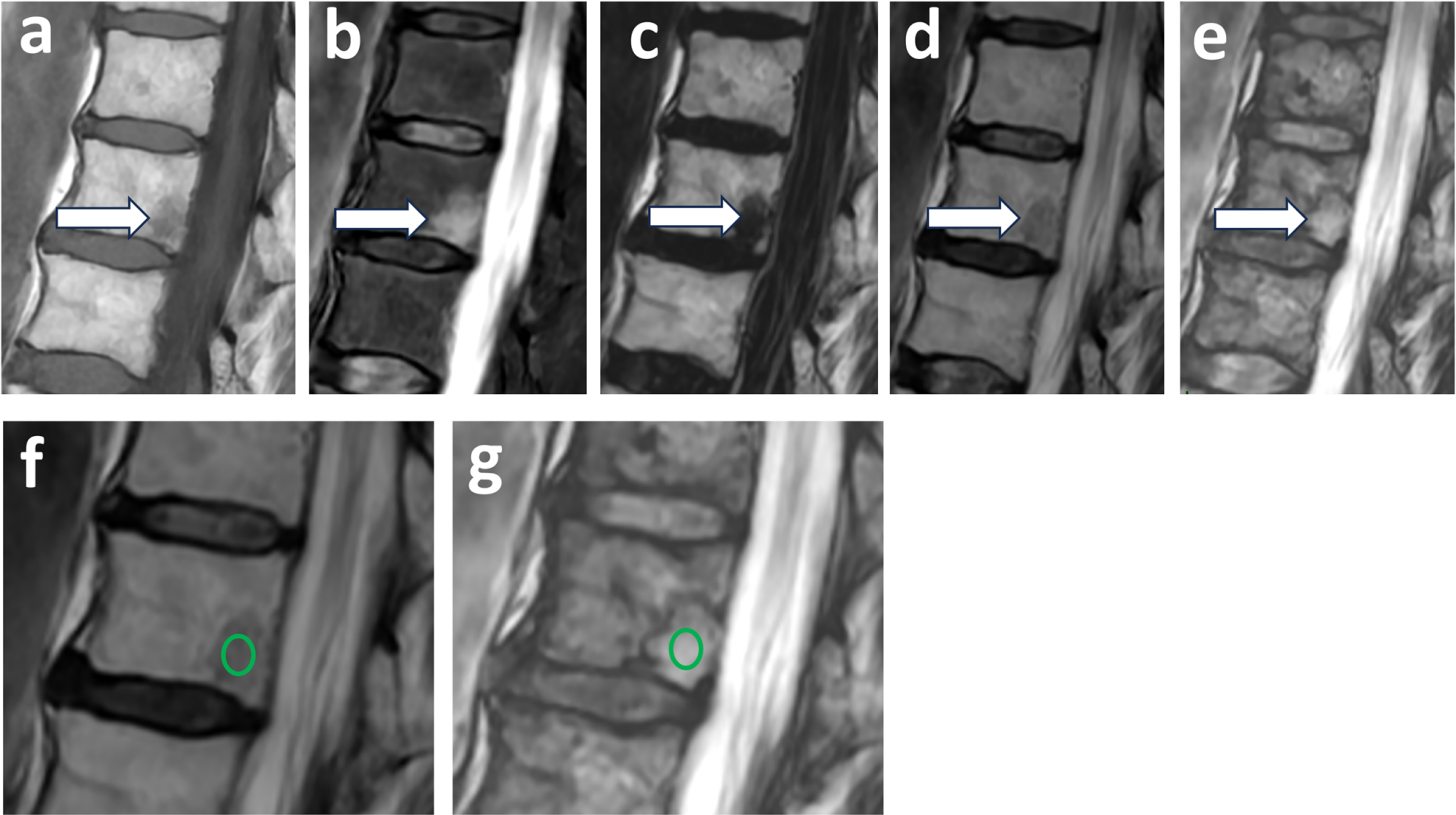
Differential diagnosis between metastasis and benign nodular marrow hyperplasia. Metastasis in a 72-year-old male with newly diagnosed prostate cancer. Sagittal spin echo T1-weighted (**a**), and fast spin echo T2 Dixon water-only (**b**), fat-only (**c**), in-phase (**d**, **f**) and out-phase (**e**, **g**) images. A focal lesion (arrows) shows low signal on T1 (**a**) and fat Dixon (**c**) images, high signal on water-only Dixon (**b**). The lesion signal shows no significant signal drop (1.3%) on the out-phase image (arrow in **e**; ROI with measured signal intensity in **g** = 588) compared to the in-phase image (arrow in **d**; ROI with measured signal intensity in **f** = 596), indicating the disappearance of marrow fat due to its replacement by tumoral tissue. These characteristics allow the confident diagnosis of a bone marrow metastasis. The diagnosis was confirmed by a lesion increase in size and the appearance of new metastases at imaging follow-up

Fluid-sensitive “T2-like” sequences are essential for lesion detection. They must be acquired with fat signal suppression to maximize contrast between lesions and marrow. Prefer FSE T2-weighted Dixon sequences over STIR or spectral fat saturation, as they generate very useful multiple image sets [22]. A single FSE T2 Dixon acquisition provides in-phase (IP), out-of-phase (OP), water-only (WO), and fat-only (FO) images. T2-weighted WO images optimize lesion detection, IP images provide anatomical detail, and IP/OP images are particularly useful for tissue characterization. A significant drop in signal intensity on the OP image compared to the IP image confirms the benign nature of lesions with mixed fat/water content (Fig. 4). Several studies have addressed the definition of this threshold, using different magnetic field strengths, GRE or FSE techniques, and T1 or T2-weighted Dixon sequences. Most of them have proposed a 20% signal drop as the threshold of significance in favor of benign conditions, although some more “careful” approaches recommend setting this threshold at 25 or 30% [23–25]. An inferior or absent drop suggests malignant marrow-replacing lesions, unless the lesion is fully fatty (See below) (Fig. 5).

The Dixon technique also forms the basis for generating FF maps which are calculated from multipoint GRE Dixon acquisitions and provide the percentage of fat present within a defined area (marrow or lesion) (Fig. 3). Whole-body coverage with a single FSE T2 Dixon sequence offers morphological information on the skeleton, but also on lymph nodes and visceral organs and allows an all-organ lesion screening [26]. Use FSE 3D T2 Dixon acquisitions when available to allow multiplanar reformations (Fig. 2).

### Recommendation # 3: Integrate DWI for functional evaluation and response assessment

Use fat-suppressed DWI sequences, typically with two b-values (e.g., 50 and 800–1000 s/mm²). DWI sequences evaluate the movements or diffusivity of water molecules within tissues, and from there tissue cellularity and integrity. Normal bone marrow has an intrinsically limited water diffusivity due to its composition of large cells, limited extracellular spaces, and predominant hydrophobic fatty content. Tumoral bone marrow replacement causes a focal or diffuse increase in water diffusivity, resulting in high signal intensity on fat-suppressed high *b*-value images and higher ADC values (0.7–1.2 × 10⁻³ mm²/s) than the normal bone marrow (0.2–0.650 × 10^−3^ mm^2^/s) [27, 28].

### Recommendation # 4: Provide quantitative information

Monitor ADC and FF during follow-up (Fig. 3). An increase in ADC and FF suggests treatment response, while a decrease suggests progression or recurrence [29]. These parameters are particularly useful when morphological sequences show stable findings, but clinical or biochemical characteristics suggest disease activity. These parameters should, however, be used with caution, as some overlap may exist between normal and pathological values, and as the values may be affected by changes in marrow cellularity following (stimulating) treatment, lesion mineralization, or fracture-related changes. Quantitative analysis should always be interpreted in conjunction with morphological findings, as part of a comprehensive “multiparametric” assessment [30].

### Remark concerning intravenous contrast injection

Contrast material injection and dynamic-contrast-enhanced (DCE) sequences should not be systematic in AS-MRI and WB-MRI protocols. Tailored and contrast-enhanced sequences may be obtained for specific organs (e.g., the brain or liver) in specific cases, depending on the preferential tropism of metastases from a given primary cancer [6]. DCE sequences can be used to assess vascularization and perfusion in lesions, which can be used to evaluate angiogenesis in MM, therapeutic response to antiangiogenic drugs, and distinguish active from inactive disease in ambiguous cases.

**Table 3:** Proposed AS-MRI and WB-MRI protocols for bone marrow study in oncology

AXIAL SKELETON MRI PROTOCOL		
Type	Plane	Anatomic coverage
T1 fast spin echo (FSE) + STIR	Sagittal	Spine
**AND**		
T1 (FSE) + STIR	Coronal	Pelvis+proximal femurs
**Or**		
T2 DIXON (FSE)	Sagittal	Spine
**AND**		
T2 DIXON (FSE)	Coronal	Pelvis+proximal femurs

WHOLE BODY MRI PROTOCOL		
Morphological sequences		
Type	Plane	Anatomic coverage
T1 (FSE or GRE DIXON)	Coronal or axial	Head- Mid-thigh
T1 (FSE or GRE DIXON)	Sagittal	Spine
**AND**		
STIR	Coronal	Head- Mid-thigh
STIR	Sagittal	Spine
	Alternative: 3D FSE T1, 3D FSE STIR	
**AND**		
T2 (FSE) for node/visceral study	Axial	Head- Mid-thigh
**AND**		
DWI	Axial	Head- Mid-thigh
Minimum 2 b-values (50–100; 800–1000s/mm²)	Multiplanar reformats (MPR) images at 2 b-values	
Optional 3-4 b values	Maximum intensity projections (MIP) high b values	
	Contrast scale inversion	

Or		
T2 DIXON (FSE)	Coronal	Head- Mid-thigh
**AND**		
T2 DIXON (FSE)	Sagittal	Spine
	Alternative: 3D T2 Dixon	
**AND**		
DWI	Axial	Head- Mid-thigh
Minimum 2 b values (50–100; 800–1000s/mm²)	Multiplanar reformats (MPR) images at 2 b-values	
Optional 3-4 b-values	Maximum intensity projections (MIP) high b images	
	Contrast scale inversion	

AND		
Quantitative biomarkers		
Type	Plane	Anatomic coverage
Fat fraction (FF) maps	Coronal or axial	Head- Mid-thigh
Apparent diffusion coefficient (ADC) maps	Coronal or axial	Head- Mid-thigh

*ADC* apparent diffusion coefficient, *DWI* diffusion-weighted imaging, *FSE* fast spin echo, *FF* fat fraction, *GRE* gradient echo, *MIP* maximum intensity projections, *MPR* multiplanar reformat, *STIR* short tau inversion recovery

## Choice of anatomical coverage and acquisition plane (Table 3)

MRI protocols for oncologic bone marrow imaging focus on areas rich in red (hematopoietic) marrow, which are preferentially involved in metastatic disease and MM.

### Option 1. Use AS-MRI for fast diagnostic and follow-up examinations

AS-MRI targets the spine, pelvic bones, and proximal femurs [1]. Covering the pelvis is important as spine-only protocols miss a significant proportion of lesions [31]. This compact protocol effectively screens a large portion of red marrow-containing areas and is cost-effective in MM and bone-predominant metastatic disease. It combines sagittal sequences for the spine and coronal sequences for the pelvis, using either a T1 plus STIR combination or a T2 Dixon sequence [32] (Fig. 1).

### Option 2. Implement WB-MRI for skeletal and “one-step all-organ” screening

“WB-MRI” usually covers the skeleton from head to mid-thighs using adapted phased-array surface coils within ~30–50 min, without contrast material injection in most cases. Breath-hold acquisitions can be used on the chest and abdomen [33].

Guideline-recommended tumor-specific WB-MRI protocols should be used for acquisition, interpretation and response assessment (e.g., Myeloma, Prostate, Breast) [34, 35]. T1 plus STIR or T2 Dixon as a single sequence can be acquired in the coronal plane or in 3D mode if available to speed up acquisition and allow multiplanar reconstructions—especially crucial for spinal and visceral assessments [26, 36]. DWI sequences expand diagnostic reach beyond bones to soft tissues, lymph nodes and visceral organs. Quantitative Dixon-derived FF maps and DWI-derived ADC maps allow quantitative assessment of response (Figs. 2, 3). If available, implement AI-based reconstruction and denoising tools to accelerate acquisition while preserving diagnostic quality.

## Lesion appearance and patterns of malignant marrow replacement

Bone marrow replacement by cancer cells may show distinct patterns on MRI, which reflect the pathophysiology of tumor spread. Focal lesions are the most frequent early manifestation in metastases and MM. Diffuse marrow infiltration suggests extensive disease, often associated with poor prognosis and increased fracture risk [37]. Two additional patterns are specific to MM: a “salt-and-pepper” appearance often seen in early disease, and a mixed focal and diffuse pattern seen in advanced cases.

On fat-sensitive T1-weighted sequences, malignant marrow-replacing lesions have low signal intensity, becoming hypointense relative to intervertebral discs and muscle. On Dixon FO images, they present as a signal void. On fluid-sensitive sequences, the signal intensity varies depending on lesion composition: higher if lytic or hydrated, lower if sclerotic or fibrous. On DWI, tumoral replacement shows high signal intensity on high b-value images and elevated ADC values, contrasting with the normal marrow [27].

## Mimics and pitfalls

Not all focal or diffuse marrow signal alterations are malignant. Benign lesions, normal variants, and therapy-related changes may mimic oncologic disease. Accurate characterization relies on meticulous analysis of information derived from morphologic and functional sequences and clinical context.

### Focal mimics of malignancy

The demonstration of the presence of fat within lesions, sometimes in even subtle quantity, is key for the correct identification of benign lesions such as richly vascular angiomas, focal nodular marrow hyperplasia, benign vertebral fractures and bone involvement by Paget disease (Fig. 4). Benign vertebral or pelvic fractures may be difficult to differentiate from malignant/pathologic fractures. Benign enostosis and benign notochordal cell tumors should also be distinguished from malignant lesions. Table 4 overviews these mimics and keys of the differential diagnosis from malignant lesions, as described in the literature [38–43].

**Table 4:** Most frequent focal mimics of malignancy and diagnostic clues for characterizing them

Mimics of tumors	Signal characteristics	Keys of correct diagnosis
Focal marrow hyperplasia	Relatively low on T1 (higher than disks/muscles) Low on T2 Relatively high on STIR/T2 WO	Preservation of fat on Dixon Fat-Only (FO) Signal drop on Dixon out-phase (OP) compared to in-phase (IP) CT may show subtle sclerosis
Vertebral hemangioma	Usually high on T1 Low T1 if atypical, predominant vessels High on T2 Often visible thick trabeculae	Preservation of fat on Dixon FO Signal drop on Dixon OP compared to IP ADC usually > 0.872 × 10⁻³ mm²/s CT may be useful (thickened vertical trabeculae)
Paget disease	Normal Sometimes high on T1 (involutive) Can be low on T1 and T2 (sclerotic) Possible relatively high T2 (fibrovascular)	Changes affect the whole vertebra Intralesional fat on MRI Bone expansion/remodeling on CT
Benign fracture	Band-like organization, parallel to the endplate Low T1/high STIR T2 Dixon Sometimes fracture line or callus visible	Preservation of fat on Dixon FO Signal drop on OP compared to IP No/subtle/non nodular soft tissue mass
Enostosis (bone island)	Signal void on all sequences Sharp or spiculated margins	No peripheral cellular “halo” on MRI (Almost) Stable over time Higher density on CT than untreated osteoclerotic metastasis
Benign notochordal cell tumor	Low T1 High on T2	Typical midline axial location No progression over time No enhancement Slightly sclerotic (no lysis!) on CT

*IP* in-phase, *OP* out-of-phase, *FO* fat only, *WO* water only (the 4 types of images generated by a single Dixon acquisition)

### Additional pitfalls

Marrow hyperplasia may be diffuse, resulting from anemia, chronic disease, or drug-induced marrow stimulation. It leads to signal loss of the bone marrow on fat-sensitive sequences resembling tumoral replacement and potentially hiding true neoplastic lesions [44]. Awareness of recent administration of marrow-stimulating factors (G-CSF and others) is key. On T2 and STIR, benign hyperplasia typically shows a uniform low signal, unlike diffuse malignancy, which shows a heterogeneous or high signal. In this scenario, Dixon imaging is valuable, as the demonstration of residual fat, even subtle, typically argues against malignancy.

Lesions with intrinsic high signal on T1, such as melanin-rich metastases of melanoma or proteinaceous MM lesions, may be overlooked on T1-weighted images due to signal similarity with the surrounding marrow. These foci are readily detected on Dixon FO and WO images or on high b-value DWI [32].

Some benign lesions (hemangiomas, degenerative disk disease, osteoporotic fractures, focal marrow hyperplasia,…) may resemble malignancy on high b-value DWI images [45]. However, Dixon FO will reveal fat, and ADC maps will show higher ADC values in these lesions than in tumors [41]. False-negative DWI findings may be present in sclerotic metastases or hypercellular red marrow. In these situations, morphologic sequences and clinical context are essential.

### Indeterminate lesions

If doubt remains regarding a marrow abnormality, comparison with prior imaging is critical. Any newly appearing lesion is highly suspicious for malignancy. If prior imaging is unavailable, consider extending MRI to additional skeletal regions (metastases and MM foci are often multiple), repeating imaging after a short interval (to see if the lesion is evolving), or performing a targeted CT to identify typical features (e.g., thickened trabeculae in hemangioma, high density in enostosis, or typical bone changes in Paget disease). Finally, a biopsy may be necessary when imaging remains inconclusive.

## Assessment of response to treatment

MRI should be the standard technique for monitoring treatment response. Unlike radiographs, CT, or bone scintigraphy, which have low sensitivity and may be confounded by phenomena such as treatment-induced sclerosis or flare, MRI allows reliable assessment of treatment effects.

As a major preliminary remark, it is essential to emphasize the importance of using the same technique and the same scanner as at baseline for all follow-up examinations, particularly when choosing between axial skeleton MRI and whole-body MRI. Similarly, if PET/CT was previously used for lesion detection, it should be used for response assessment.

### Recommendation 1: Perform visual analysis of morphological and DWI sequences

**Fig. 6:**
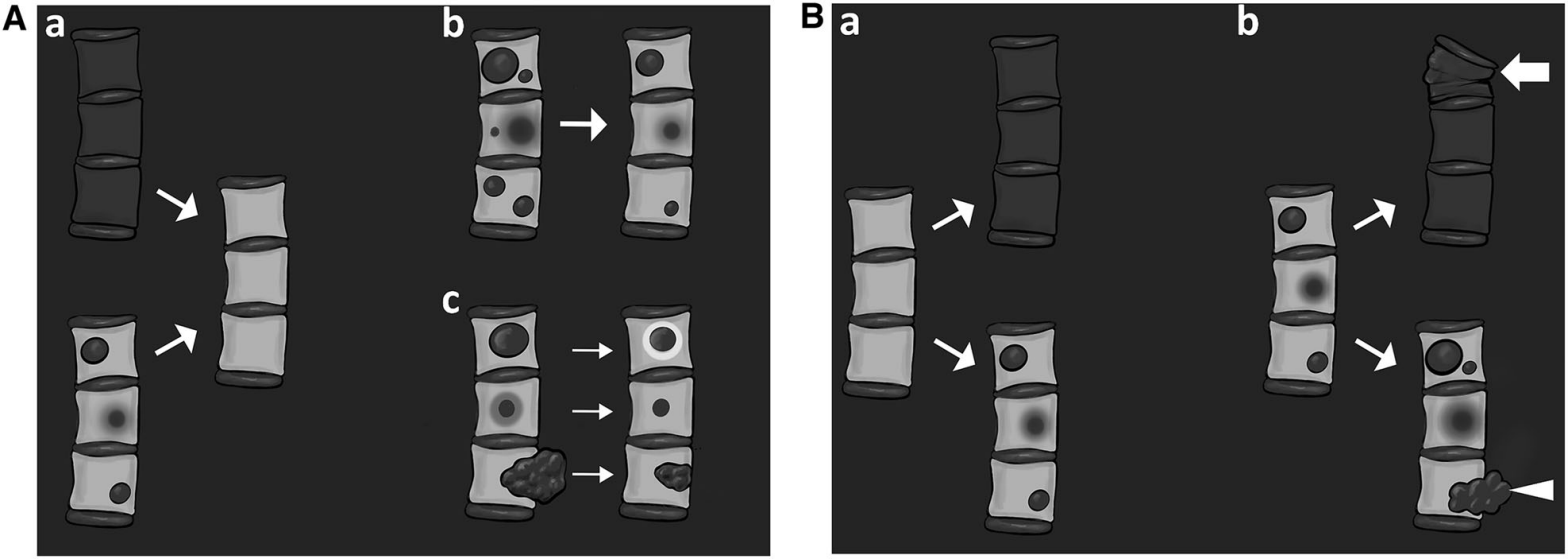
Schematic representation of response assessment on morphologic T1-weighted or high b-value DWI sequences (inverted grayscale). **A** Signs of tumor response in metastatic disease or myeloma. **a** Evolution from diffuse or focal neoplastic involvement to a normal marrow appearance indicates complete response. **b** Decrease in lesion size and number indicates partial response. **c** Ancillary signs indicative of response on follow-up T1-weighted MR images: appearance of a peripheral fatty halo surrounding a lesion (top vertebra), disappearance of a peripheral cellular low signal intensity halo (middle vertebra), and a decrease or disappearance of soft tissue involvement (bottom vertebra) indicate a responsive lesion. **B** Signs of disease progression. **a** Evolution from normal-appearing marrow to focal or diffuse marrow infiltration patterns indicates disease progression. **b** Evolution from focal to diffuse marrow involvement (upper part) or an increase in lesion size and number (lower part) indicates progressive disease. Appearance of a pathologic (vertebral) fracture (upper vertebra, arrow) and appearance/increase of soft tissue extension (lower vertebra, arrowhead) also indicate disease progression

Begin by evaluating the number, size, and appearance of lesions on morphological sequences (T1, STIR, Dixon FO and WO, high b-value DWI images). Follow the number and size to assign lesions to response categories such as complete response, partial response, stable disease, or progression, mirroring RECIST-like methodology [1] (Fig. 6).

Specific imaging signs, such as a “fatty halo” appearing at the periphery of a treated lesion, or the loss of a hyperintense rim (“cellular halo”) seen on pre-treatment water-sensitive fat-suppressed sequences, are reliable signs of response [46] (Fig. 6).

Responding lesions typically show decreased signal on high b-value DWI.

### Recommendation 2: Incorporate quantitative analysis using ADC and FF

Quantitative metrics provide early and objective evidence of treatment response. Measure ADC and FF within target lesions at baseline and at follow-up, using carefully positioned ROIs in a similar way each time. An early increase in lesion ADC and FF supports treatment response, while an early decrease in either suggests progression [29] (Fig. 3). A 25% ADC increase can be used as the cut-off value for suggesting a response [34, 35]. Later on, after treatment response, reversal to normal marrow leads to a decrease in ADC [47].

ADC measurements allow differentiation of viable tumors (which have lower ADC values) from necrotic metastases or treated myeloma foci (which may have high signal on high *b*-value images due to the “T2 shine through” phenomenon, but have much higher ADC values than viable tumors).

### Recommendation 3: Use standardized response criteria and reporting

Whenever possible, apply validated reporting systems that integrate morphological and functional criteria, and have been supported by histopathology and survival outcomes in metastatic disease and MM [6, 29, 34, 35]. Reports should include a baseline summary, description of imaging biomarkers used (e.g., DWI, ADC), and clear categorization of response for each evaluated site (response, stability or progression under treatment). Consistent longitudinal imaging and harmonized reporting terminology are critical for guiding oncologic management.

## Complications of bone marrow lesions and treatment-related changes

### Identify disease-related skeletal complications

Radiologists should systematically evaluate the skeleton and especially the spine for signs of complications related to bone involvement by cancer. Pathological fractures and neurological complications are frequent in metastatic disease and MM [48].

MRI is the modality of choice for detecting malignant fractures, epidural extension, and cord compression [49]. Native or reconstructed sagittal and transverse images should be obtained. Malignant fractures often show diffuse signal changes, accompanying soft tissue mass, and disappearance of fat. Benign osteoporotic fractures typically demonstrate preserved marrow fat and visible fracture lines or callus formation. When bone detail or better evaluation of the bone strength is needed for treatment, a supplemental CT may be used.

### Recognize marrow changes induced by treatment

Post-treatment marrow changes must be distinguished from active disease. Accurate MRI interpretation requires thorough knowledge of the clinical history and the treatment timing [50].

Radiotherapy initially causes geographic marrow edema, followed by fatty replacement in irradiated segments. The treated areas appear hyperintense on fat-sensitive images and hypointense on STIR or water-only sequences, often with sharp margins (Fig. 1).

High-dose chemotherapy typically induces diffuse marrow hypoplasia, characterized by uniform signal increase on fat-sensitive sequences.

Steroid therapy increases the risk of osteonecrosis, especially in epiphyses. Look for serpiginous margins with low signal on T1 and high signal on T2/STIR. Monitor for signs of collapse in load-bearing regions.

## Summary statement

Bone marrow MRI plays an essential role in the oncologic imaging pathway and should be implemented for diagnosis, staging, response evaluation, and follow-up in both metastatic bone disease and MM. It offers superior sensitivity over radiographs, CT, and bone scintigraphy and provides radiation-free, whole-skeleton assessment with high tissue contrast.

MRI is now recommended alongside PET/CT for staging and assessment of treatment response, with the advantage of tracer independence and absence of radiation.

While AS-MRI is an effective approach in many routine situations for disease staging and follow-up, WB-MRI is preferred when comprehensive staging is needed. WB-MRI protocols should include both morphological fat-sensitive and fluid-sensitive sequences and functional DWI sequences. Sequence selection and reporting should follow tumor-specific guidelines and incorporate quantitative ADC and FF mapping for treatment monitoring.

Radiologists must be familiar with the typical patterns of bone involvement, the spectrum of benign mimics and pitfalls, and therapy-related marrow changes to avoid misdiagnosis. These practical recommendations are designed to help radiologists integrate bone marrow MRI into routine oncologic practice.

## Patient summary

MRI offers direct, non-irradiating visualization of the bone marrow, usually without contrast injection.

MRI provides an accurate and early way to detect cancer in the bone marrow.

It supports proper staging of the disease, treatment planning, and follow-up to evaluate response.

MRI can be repeated over time to monitor for recurrence or treatment-related complications.

## Supplementary information

ELECTRONIC SUPPLEMENTARY MATERIAL

## Abbreviations

ADC: Apparent diffusion coefficient
AS-MRI: MRI of the axial skeleton (spine, pelvis, proximal femurs)
DWI: Diffusion-weighted imaging
FF: Fat fraction
FO: Fat only Dixon image
FSE: Fast spin echo
GRE: Gradient echo
IP: In-phase Dixon image
MM: Multiple myeloma
OP: Out-of-phase Dixon image
PET/CT: Positron-emission tomography with computed tomography
STIR: Short tau inversion recovery
WB-MRI: Whole-body MRI
WO: Water only Dixon image
