# Supplementary material for: ESR Essentials: bone marrow MRI in oncology—practice recommendations by the European Society of Musculoskeletal Radiology
Source: Eur Radiol. 2026 Feb 9;36(7):5414–28. doi: 10.1007/s00330-025-12307-4 (PMC13282236; doi:10.1007/s00330-025-12307-4)
Supplement: Supplementary file 1 — ELECTRONIC SUPPLEMENTARY MATERIAL [file 330_2025_12307_MOESM1_ESM.pdf]

ESR Essentials: bone marrow MRI in oncology - practice recommendations by the European Society of Musculoskeletal Radiology

Electronic Supplementary Material

Clinical Reporting Template for Bone Marrow MRI

Table illustrating the proposed structured reporting for bone marrow MRI studies performed in metastatic cancer or myeloma, both for axial skeleton (AS-MRI) and whole body (WB-MRI) approaches.

Section	Axial Skeleton MRI (AS-MRI)	Whole Body MRI (WB-MRI)
**Indication**	Clinical context (staging, follow-up), symptoms, treatment	Clinical context (staging, follow-up), symptoms, treatment
**Technique**	• Magnet/field<br>• Covered areas<br>• Sequences<br>• Protocol modifications	• Magnet/field<br>• Covered areas<br>• Sequences<br>• Protocol modifications
**Comparison**	• Date of previous AS-MRI<br>• Other examinations available	• Date of previous WB-MRI<br>• Other examinations available
**Bone Marrow Evaluation**	• Marrow infiltration patterns (normal, focal, focal on diffuse, diffuse, micronodular)<br>• Involved skeletal areas: spine (cervical → lumbar), pelvis, proximal femurs	• Marrow infiltration (normal, focal, focal on diffuse, diffuse, micronodular)<br>• Involved skeletal areas: skull, spine (cervical → lumbar), scapular girdle, pelvis, ribs, sternum, long bones, other regions
**Quantification of focal bone lesions**<br>**Morphology: size, number**<br>**Biomarkers**	• Choose up to 5 bone lesions<br>• Largest diameter	• Choose up to 5 bone lesions<br>• Largest diameter<br>• Measure FF and ADC in up to 5 lesions.
**Fractures**	• Presence<br>• Location<br>• Benign vs malignant based on morphology<br>• Risk/mass effect (spine, cord, …)	• Presence<br>• Location<br>• Benign vs malignant based on morphology and quantitative parameters<br>• Risk/mass effect (spine, cord, …)
**Extraosseous evaluation**	Extraosseous/ extramedullary disease	• Systematic organ screening<br>• Lungs, liver, nodes, other
**Quantification of extraosseous lesions**<br>**Morphology: size, number**<br>**Biomarkers**	• Lesion size	• Choose up to 5 extraosseous lesions<br>• Lesion size<br>• Measure ADC in up to 5 lesions
**Treatment Response Assessment Category (RAC)**	• NA if baseline<br>• Response / Stable / Progression	• NA if baseline<br>• Response / Stable / Progression
**Suggest a site for biopsy if necessary**	………..	………
**Incidental Findings**	Report incidental/treatment-related findings (avascular necrosis, other neoplasms, others)	Report incidental/treatment-related findings (avascular necrosis, other neoplasms, others)
**Conclusion**	• Main lesions location<br>• Response Assessment Category<br>• Incidental findings	• Main bone/extraosseous lesions location<br>• Response Assessment Category<br>• Incidental findings
